# Making Sense of “Nonsense” and More: Challenges and Opportunities in the Genetic Code Expansion, in the World of tRNA Modifications

**DOI:** 10.3390/ijms23020938

**Published:** 2022-01-15

**Authors:** Olubodun Michael Lateef, Michael Olawale Akintubosun, Olamide Tosin Olaoba, Sunday Ocholi Samson, Malgorzata Adamczyk

**Affiliations:** 1 Faculty of Chemistry, Warsaw University of Technology, 00-664 Warsaw, Poland; lateefolubodun@gmail.com (O.M.L.); michaelakintubosun@gmail.com (M.O.A.); sunday.samson@pwr.edu.pl (S.O.S.); 2 Laboratory of Functional and Structural Biochemistry, Federal University of Sao Carlos, Sao Carlos 13565-905, SP, Brazil; olaobamide@gmail.com

**Keywords:** genetic code, orthogonality, synthetic biology, amber suppressors, frameshift suppressors, tRNA modifications, metabolism

## Abstract

The evolutional development of the RNA translation process that leads to protein synthesis based on naturally occurring amino acids has its continuation via synthetic biology, the so-called rational bioengineering. Genetic code expansion (GCE) explores beyond the natural translational processes to further enhance the structural properties and augment the functionality of a wide range of proteins. Prokaryotic and eukaryotic ribosomal machinery have been proven to accept engineered tRNAs from orthogonal organisms to efficiently incorporate noncanonical amino acids (ncAAs) with rationally designed side chains. These side chains can be reactive or functional groups, which can be extensively utilized in biochemical, biophysical, and cellular studies. Genetic code extension offers the contingency of introducing more than one ncAA into protein through frameshift suppression, multi-site-specific incorporation of ncAAs, thereby increasing the vast number of possible applications. However, different mediating factors reduce the yield and efficiency of ncAA incorporation into synthetic proteins. In this review, we comment on the recent advancements in genetic code expansion to signify the relevance of systems biology in improving ncAA incorporation efficiency. We discuss the emerging impact of tRNA modifications and metabolism in protein design. We also provide examples of the latest successful accomplishments in synthetic protein therapeutics and show how codon expansion has been employed in various scientific and biotechnological applications.

## 1. Introduction

**Figure 1:**
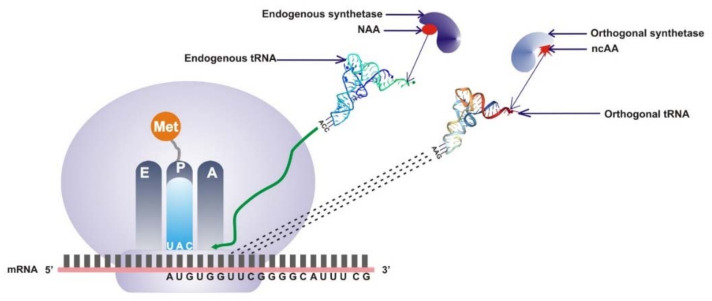
Schematic illustration of the general principles for the site-specific incorporation of ncAA into proteins in vivo. The natural translational system involves the activity of endogenous synthetase, which charges the endogenous tRNA with natural amino acids (NAAs), which is presented as a red oval shape. The orthogonal translational system is applied to site-specifically incorporate ncAA into proteins. Orthogonal aminoacyl-tRNA synthetase acylates the orthogonal tRNA with a noncanonical amino acid. The acylated orthogonal tRNA introduces the ncAA at a specific site complementary to the unique codon, thereby incorporating it into the ORF that encodes the protein of interest (ncAA is represented by the red star).

Genetic code expansion (GCE) is a powerful synthetic biology tool that is used to drive the site-specific incorporation of noncanonical amino acids (ncAAs) into any protein of interest in cells. The chemical diversity of the building blocks can further be explored beyond the canonical usage of 20 standard amino acids during protein synthesis, thereby significantly improving the functions and usefulness of proteins in different biochemical systems. The translational system consists of the ribosome, which is the cellular factory of protein synthesis; amino acids; and tRNA/aminoacyl-tRNA synthetase (aaRS) pairs. A synthetase charges the tRNA with cognate amino acids to form aminoacyl-tRNA via a two-step ATP energy-consuming reaction. Genetic information is encoded by 64 triplet codons comprising 61 sense codons that code for amino acids and 3 nonsense codons responsible for the termination of protein biosynthesis. Because these 3 codons do not specify amino acids, they are called nonsense codons or stop codons, and they include UAG (amber), UGA (opal), and UAA (ochre) [1]. The three successive stages of protein synthesis in the ribosomes include initiation, elongation, and termination [2]. The initiation stage is marked by the assembly of the translation initiation complex and the suppression of the start codon AUG by methionine-charged tRNA (Figure 1). The movement of methionine-charged tRNA to the P site causes a conformational change that allows another charged tRNA to bind to the A site of the large ribosomal subunit. The elongation stage involves the activation of the elongation factor EF-Tu (elongation factor translation thermo unstable) and a GTPase, which catalyzes the formation of peptide bonds in the growing peptide complex of amino acids on the P site. Specifically, elongation progression depends on the cognate match pair of amino acids and tRNAs, a process known as charging and characterized by specific aminoacyl-tRNA synthetases (aaRSs) [3]. Unloaded tRNA leaves the ribosome through the E site, and newly charged tRNA enters at the A site. The translational process is terminated when the ribosome reaches the stop codon. Because the stop codons do not code for any amino acids, the release factor RF-1 (which recognizes UAA and UAG) or RF-2 (which recognizes UAA and UGA) competes for the stop codon. Consequently, the release factors hydrolyze the peptidyl-tRNA, and the polypeptide is released [4,5].

The use of a canonical genetic code with triplet codons is an important characteristic traceable to the ancestral evolvement of all living organisms [6,7], with few exceptions observed in viruses and bacteria. The reassignment of codons in ciliates and mitochondria is a form of codon usage enhancement. The reassigned codons encode for the “21st” amino acid selenocysteine (Sec), which is now being used for their regulatory mechanisms by the cellular protection against oxidative stress and in the synthesis of growth hormone [7,8], and the “22nd” amino acid pyrrolysine in methanogenic archaea and bacteria is encoded by UAG (stop codon) [9].

From the synthetic biology point of view, the canonical usage of genetic codes specifies amino acids with a limited number of functional groups, such as thiol ether, amides, and amines and alkyl and aryl groups, which all suffice the naturally evolved organisms; however, these canonical codons are not sufficient to exhaustively incorporate ncAAs. Therefore, a synthetic biology approach involving the application of a genetic code extension as a bioengineering tool has been utilized for two decades to further improve and overcome the limitations of incorporating nonstandard amino acids into proteins. The contribution of the multiple factors affecting incorporation efficiency is largely unknown. Some of these limitations at least in amber stop codon reassignment include the competition of suppressor tRNAs with release factors, which reduces suppression efficiency and the limited number of non-natural amino acids that can be incorporated into one protein at a time [10].

An approach to enhancing the incorporation of ncAA includes the use of an orthogonal pair [1] that encompasses engineered tRNA and aminoacyl-tRNA synthetase (aaRS) redesigned to deliver a dedicated ncAA to the ribosome [11,12]. tRNAs, as an indispensable component of the GCE tool, are modified by the host cell metabolism. Modifications of tRNA contribute to translational fidelity in vivo [13] and may also affect the recognition of the extended four-base codons in genetically manipulated mRNAs. Furthermore, a plethora of chemical modifications presented on tRNA have been reported to affect tRNA/aaRS pair-specific interaction and may contribute to tRNA stability [14]. New steady-state levels of tRNA molecules, synthetized due to the introduction of a multicopy array of orthogonal pairs into the host cell, are expected to perturb the host cell homeostasis by breaking the balance between the total tRNA pool and tRNA modifying enzymes. This is further followed by remodeling the host cell metabolism to a new steady state, for which the exact intracellular clues are not well known, and the specific triggers are not yet defined. We show in this review article how modification of tRNAs can have an impact on the translational fidelity, incorporation efficiency of ncAAs into the host cell, and host metabolic homeostasis itself. The o-tRNA often used for GCE requires full orthogonality to avoid interactions with host modification and to enable processing enzymes to become functional, which is also vital for cellular fitness. This article also reviews the advancements in codon expansion and discusses how the efficiency of ncAA incorporation can further be enhanced by remodeling the host cell metabolism.

## 2. The Principles of Engineering the Genetic Codes

### 2.1. Directed Evolution of Orthogonal Translational System of Expanded Codons

The creation of the natural orthogonal system was first described in 2002 when it was used for the site-specific incorporation of a nonstandard amino acid pyrrolysine, which was then referred to as the 22nd amino acid [15,16], into the proteome of living cells with the use of an amber stop codon. The common method employed in orthogonal translation systems (OTSs) includes the use of a natural amber suppressor known as pyrrolysyl-RS (PylRS)/tRNA^Pyl^ pair, which is derived from *Methanosarcina barkeri* [17], and tyrosyl-RS (TyrRS)/tRNA^Tyr^ derived from *Methanococcus jannaschii.* The pyrrolysine system has shown great orthogonality in both prokaryotes and eukaryotic cells. Such orthogonality has enabled the evolution of PylRS/tRNA^Pyl^ pairs in *E. coli*, and the evolved PylRSs have been applied to site-specifically incorporate ncAAs into proteins in *Drosophila melanogaster*, *Caenorhabditis elegans*, and *Saccharomyces cerevisiae* and to add L-lysine derivatives to the genetic codes of mammalian cells [16,18,19,20]. Till date, over 200 ncAAs have been derivatized and site-directedly [21,22] incorporated into protein [1,23].

**Figure 2:**
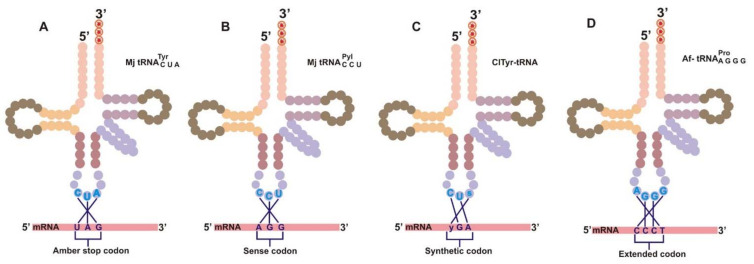
(**A**) Schematic illustration of the different approaches that are used for the incorporation of ncAA. (**A**) Amber codon suppression. (**B**) Rare sense codon reassignment. (**C**) Triplet codon and anticodon composed of natural and synthetic bases. (**D**) Frameshift suppression with the use of extended codon (e.g., a quadruplet codon).

The overall mechanism of codon expansion through directed evolution encompasses the construction of a mutant library with tRNAs and aaRS derived from a source organism. Since in most cases aaRS and tRNA are synthesized from another domain of life with limited evolutionary relatedness, the pairs are orthogonal to the host cells’ translational machinery [24,25,26,27]. Orthogonal tRNA (o-tRNA) is designed such that the endogenous aaRS of the host does not charge it with natural amino acid but shows specificity towards a given ncAA; thus it is not perturbed in its function during translation in the orthogonal system. This is achieved when o-tRNA is specifically aminoacylated by an orthogonal aminoacyl-tRNA synthetase. tRNA is uniquely designed to decode any of the reassigned nonsense codons (UAG/UAA/UGA) or sense or extended codons (Figure 2). After incorporating the ncAA, tRNA charged with ncAA is recognized by the ribosome, and it allows the ncAA to be site-specifically incorporated into the polypeptide chain during translation.

Orthogonal translational systems (OTSs) have been shown to be efficient in encoding natural amino acids [27,28] and thus can be functionally engineered to play the role of a cognate system to the one with natural tRNA/aaRS pairs (Table 1). Consequently, these engineered tRNA/aaRS pairs have been repurposed for genetic code expansion [29,30]. The repurposing of aaRS specificities towards the TyrRS/tRNA_CUA_^Tyr^ pair from *M. jannaschii* was first reported by Wang and Schultz [24]. Recent advancements have offered new methodologies in molecular evolution and genome engineering that contribute to the evolved performance of OTSs in vivo. In a study by Bryson et al. [31], phage-assisted continuous evolution (PACE) was used to generate active PylRS variants with improved activity by 45-fold and *Methanocaldococcus jannaschii* tyrosyl-tRNA synthetase (MjRS) variants showing enhanced specificity [31]. Most OTSs are placed on plasmid vectors; however, Amiram et al. [32] reported the use of multiplex automated genome engineering (MAGE) in a genomically recoded organism (GRO) to produce chromosomally encoded MjRS enzymes with activity increased by 25-fold, which resulted in the ability of the system to produce a single protein containing as many as 30 ncAAs at high yield [32]. Further efforts in the field of synthetic biology have been undertaken for advancing the strategies of incorporating ncAA into proteins by developing alternative strategies to engineer OTS, such as utilizing codons that can be reassigned for dedicated incorporation of ncAAs, and expanding their incorporation beyond L forms of amino acids (L-AAs), for instance, through ribosome engineering [33]. The genetic engineering of the rRNA structure enables the robust incorporation of proteins and peptides containing ncAA by intentionally modifying 23S rRNA in regions critical for peptide bond formation. The use of modified ribosomes makes it possible to directly incorporate dipeptides, β-amino acids, D-amino acids, and dipeptidomimetic analogues of the normal proteinogenic L-α-amino acids (for a detailed review, see [34]).

### 2.2. Site-Specific Incorporation of ncAA into Proteins by Engineering of the Codon Anticodon Interface

The site-specific incorporation of ncAA requires the accurate suppression of amber codon or four-base codon by its specific suppressor tRNAs. The understanding of encrypted codes by biological translators delineates the ease of translation with a diminished likelihood of misacylation. As mentioned in Section 1, the acylation of amino acid to a growing peptide chain on the ribosome is a fundamental outcome of codon–anticodon interaction between charged tRNA and mRNA on the ribosome. Through directed evolution, the interaction of nonsense codons with engineered orthogonal tRNA (o-tRNA) can be enhanced to achieve site-specific insertion of ncAA into the growing polypeptide chain [59,60,61]. An interaction between the engineered tRNA and nonsense codons is limited because of competition with termination signals, such as release factors that recognize stop codons and cause the termination of the translation process [10]. One of the major challenges to this process is the difficulty of suppressing the release factor-mediated chain termination reaction and allowing an efficient interaction between the engineered tRNA and nonsense codons. Therefore, four alternative approaches have been used to insert ncAA in a site-specific manner, including amber codon suppression, rare sense codon reassignment, nonstandard codons with synthetic nucleotide, and extended codons (Figure 2).

The most frequently used nonsense codon is the amber nonsense codon, although the use of ochre and opal has also been reported [56,62,63,64]. The least used codon among the three stop codons in *E. coli* is amber (UAG), and it can be reassigned to encode ncAAs [35,65,66] without interfering with the existing protein termination signals. This codon can be incorporated as a new insertion into the ORF or through substitution mutation of the original sense codon (Figure 2A). The efficiency of amber codon recognition by orthogonal tRNA_CUA_ can be enhanced usually by knocking out or mutating the release factor 1 (RF-1) [5,67,68], thereby increasing the efficiency of orthogonal tRNA_CUA_ to recognize the amber codon and also enhancing its competitive advantage to bind to the amber codon [35]. *E. coli* strains with all UAG recoded to the synonymous UAA codon, which permits the deletion of RF-1, are now available [68].

The usage of rare sense codon reassignment [69,70,71] for the incorporation of ncAAs was achieved by repurposing rarely used sense codons, such as AGG (Arg codon) [69,70] and AUA (Ile codon) [71], using newly designed tRNA/aaRS pairs, PylRS/tRNA_CCU_^Pyl^ and *Met*RS/tRNA_LAU_^Ile2^, respectively, in *E. coli* (Figure 2B). Additionally, Hirao et al. [72] demonstrated the use of an unnatural base pair of CUs (2-amino-6-(2-thienyl) purine, s) and yAG (pyridine-2-one, y) in the expansion of a genetic code (Figure 2C). The ribonucleoside triphosphate of pyridine-2-one was site-specifically inserted into mRNA complementary to 2-amino-6-(2-thienyl) in a template by T7 RNA polymerase. The yAG codon in the transcribed ras mRNA was recognized by the CUs anticodon of a yeast tyrosine tRNA variant. The tRNA with nonstandard anticodon CUs was aminoacylated with 3-chlorotyrosine (CITyr) in an *E. coli* cell-free system.

Quadruplet codons in manipulated mRNA encoding ncAAs have been proven to overcome the limitations associated with the use of triplet codons in the translational system [37,73,74,75,76,77]. Inserting a single-base sequentially after the triplet codon, thus creating a frameshift mutation at a specific position, makes an engineered quadruplet tRNA (tRNA_AGGG_, tRNA_UCCU_, and tRNA_CUAG_) to recognize quadruplet codons (CCCT, AGGA, and CTAG) [78,79] (Figure 2D). The quadruplet codon strategy has been shown to be more efficient and benevolent than the amber suppression approach. It also avoids the competition between suppressor tRNA and endogenous release factors. It offers the possibility of introducing more than one ncAA into a single protein by using two different and independent four-base codons on mRNA. Extending the genetic codons by one extra-base pair would increase the overall canonical triplet codons from 64 to 125. Four-base codons, such as CGGG and AGGU, are suppressed by tRNAs carrying complementary anticodons without much competition from endogenous tRNAs [8]. For example, two different ncAAs have been successfully incorporated into a single protein using CGGG and AGGU four-base codons [49,80]. These extended codons were used to incorporate an NBD derivative of lysine and 2-naphthylalanine into streptavidin in vitro with two chemically acylated frameshift suppressors. More lately, two [11,81], three [80], and five [82] ncAAs were incorporated into a single protein without compromising the yield of protein synthesis.

In a study by Magliery et al. [75], a combinatorial approach was employed to exhaustively identify tRNAs that efficiently suppress four-base codons in the β-lactamase gene as a model gene. A reporter library was constructed in which four random nucleotides replaced a serine codon in the β-lactamase gene. The suppressor library consisted of tRNA_2_^Ser^ derivatives that had an anticodon loop of seven nucleotides, including A residue at position 37 modified with 2-methyl-thio i^6^A (ms^2^i^6^A), which were replaced with either eight or nine random nucleotides to generate an o-tRNA suppressor library. Combining these two libraries led to the generation of an appropriate frameshift o-tRNA that suppressed the four-base sequence as a single codon. This strategy has also been used in the selection of novel five- and six-base codon suppressors [83]. Similarly, in the method employed by Schultz et al., engineered Pyl-tRNA_UCCU_^Pyl^ variants that were previously used to incorporate a lysine derivative, N-3-(tert-butyloxycarbonyl)-L-lysine (Boc-Lys), was used to decode AGGA codon in both *E. coli* and mammalian cells [37]. This tRNA evolution improved the acylation of tRNA_UCCU_^Pyl^ by a PylRS variant and enhanced ribosome affinity to quadruplet codons. Thus, the efficiency of a quadruplet codon decoded by the natural ribosome was optimized without further engineering the host cell translational machinery [37]. Recent published studies have been gathered on the use of quadruplet codons to encode ncAAs [33,73,74,84,85].

## 3. tRNA Modifications Affect the Synthesis of Non-Natural Proteins

Recent progress in the engineering of the codon–anticodon interface to extend the capacity of translational efficiency beyond the triplet codons has made it possible to improve incorporation efficiency by modifications of tRNA with extended anticodons. tRNA modifications play an essential role in the codon–anticodon pairing and decoding process, thereby ensuring base-pairing flexibility and expanding the ability of tRNAs to read additional codons. Identifying distinct modifications at the vicinity of o-tRNA anticodons may be a golden strategy that can be utilized to improve the synthesis of non-natural proteins. However, there is a dearth of experimental studies focusing on applying modified nucleosides of o-tRNAs with extended anticodons at the codon–anticodon interface. Therefore, further studies should be redirected towards improving the translational fidelity of non-natural proteins via modification of hypomodified nucleosides of extended anticodons of o-tRNAs to enhance the translation efficiency of ncAAs.

Structurally, tRNA is characterized by four hairpin helices and three major loops with a highly conserved 3^1^-CCA sequence at the variable loop [28]. All tRNAs consist of three regions: an acceptor end, an anticodon end, and a core region. The core region is composed of the dihydrouridine loop (D), variable loop (V), and TψC loop [86]. The anticodon stem-loop (ASL) of tRNA is hosted with the most complex and diverse chemical structures in either the anticodon at the wobble position or the anticodon position directly adjacent to it [87,88]. Many tRNAs have modifications at the first (wobble) position of the anticodon (position 34) and adjacent to the 3′-position of the anticodon (position 37). Most of the proven modifications of tRNA that occur at position 34 are essential for precise decoding by the codon–anticodon interaction and have been shown to optimize mRNA decoding function [87,89,90]. Position 37 of the tRNA usually contains a modified purine nucleoside. These modifications play a critical role in stabilizing codon–anticodon pairing through the base–stacking interactions and function to maintain the reading frame. In *E. coli* cells, 61 sense codons out of the total 64 codons present in the genome are decoded by 43 tRNA species. A total of 21 codons out of these 43 tRNAs have been identified as a potential target for reassigning of sense codons [91].

tRNA is a major class of RNA molecules that play an important regulatory role in the protein translation by transferring acylated amino acids to the ribosome. It acts as a regulator of the translation process under changing growth conditions [92,93,94] and translates the genetic code using tRNA modification tunable transcripts (MoTTs), details in Section 3.5. For this reason, the efficiency of the GCE system is dependent on the type of chosen protein models and the overall codon composition of mRNA. Over 80 modifications in the structural core of tRNA have been reported to be essential for the stabilization of the molecular structure of proteins [13,14,95], while the loss of modifications may lead to the degradation of hypomodified tRNAs. The anticodon and acceptor stems of tRNAs contain a unique nucleotide sequence called identity elements [96], which is critical to recognizing a specific tRNA by a cognate aaRS [97]. Moreover, the interaction of these identity elements with aaRS often determines the specificity/orthogonality of many tRNA/aaRS. However, the possibility of null interaction between these identity elements in tRNA and some aaRSs was earlier reported by Nozawa et al. [17]; thus, it is not uninformative to speculate a priori that the absence of such interaction is benevolent to codon reassignment.

Inchan et al. [98] were the first to report the breaking of the degeneracy of the genetic code by efficient replacement of phenylalanine (Phe) with L-3-(2-naphthyl)alanine at UUU using a mutant yeast, tRNA_AAA_^Phe^, derived from wild-type tRNA_GAA_^Phe^. The altered anticodon loop tRNA_AAA_^Phe^ allowed for exclusive recognition of UUU codon by standard Watson–Crick base pairing between codon and anticodon rather than read through by a G-U wobble base pair at the first position of the anticodon. Additionally, G37 was replaced with A in the mutated tRNA_AAA_^Phe^. It is worth noting that in eukaryotic and archaeal tRNA^Phe^, nucleotides at position 37 are hypermodified to wybutosine (yW) [99].

Other studies have also documented the potential roles of tRNA modification in incorporating ncAAs by reassigning rare serine, arginine, isoleucine [70,71,100,101,102], lysine, asparagine, phenylalanine, and histidine codons [103]. Similarly, yeast suppressor tRNA_ΨΨA_^Tyr^ recognizing exclusively ochre UAA codon has been created by the replacement of G34 at the first position of anticodon in tRNA_G__ΨA_^Tyr^ with pseudouridine Ψ34 [104]. The efficiencies of some tRNA nonsense suppressors decrease in a tRNA modification-dependent manner. For example, the translation efficiency of tRNAs from *E. coli* and *S. typhimurium* sense codons with uridine at either the first or third position, and tRNA modification deficiencies for 2-methylthio-N-6-isopentenyladenosine (ms^2^i^6^A37) or 2-methylthio-N-6-(cis-hydroxy)isopentenyladenosine (ms^2^io^6^A37) at the 3′ side of the anticodon (position 37), is dependent on the activity of MiaA and MiaE, respectively. The mutation of *miaA* reduces the efficiency of tRNA_CUA_^Ser^, tRNA_CUA_^Tyr^, and tRNA_CUA_^Leu^ suppressors [105]. Providing an overdose of certain tRNA-modifying enzymes (Table 2) might be beneficial for GCE systems for the suppression of nonsense codons in different codons’ context in vivo.

### 3.1. tRNA Processing by Intracellular Machinery May Influence Incorporation Efficiency in GCE Systems

At any point in the tRNA maturation pathway, tRNAs can be post-transcriptionally modified, which tends to influence their proper folding and functions. The modification of tRNAs can take place in different intracellular compartments, and as they travel across cellular membranes, they encounter different modification enzymes, which dictate their fate in the cell. All types of RNAs, including tRNAs, are synthetized by the same RNA polymerase in procaryotes. In *E. coli*, at the beginning of transcription, the σ^70^-subunit recognizes the -10 and -35 region of a gene promoter and recruits the core of the RNA polymerase containing five subunits: Two α, one β, one β’, and one ω [123]. The DNA strands are unwound to form a single-stranded “transcription bubble” within a catalytically active RNAP-DNA open complex [124]. Once the RNA polymerization begins, the σ-subunit dissociates, and elongation continues until it reaches a site called the terminator. However, in eukaryotes, tRNA is synthetized by RNA polymerase III (RNAP III). From yeast to mammalian cells, the evolutionary conserved regulator Maf1 negatively regulates the activity of RNAP III and modulates tRNA synthesis rates in response to growth conditions. The association of the tDNA promoter region with RNAP III is weakened when Maf1 interacts with RNAP III, which, consequently, represses tRNA transcription. Under optimal environmental growth conditions, Maf1 dissociates from RNAP III [125] and tRNA can be efficiently synthetized. Therefore, when GCE is performed in eukaryotic cells, this requires a case study for establishing optimal growth conditions.

In the maturation process, the pre-tRNA transcripts undergo post-transcriptional modifications by these enzymes that carry out their function with high fidelity. This is largely attributed to the high specificity of the enzymes to the pre-tRNA binding pocket. Additionally, the 5′ and 3′ sequences protect mature tRNA species from the multiple additions of CCA. Studies have shown that the modification of tRNAs takes place either before or after maturation in a specific order. This was observed when an intron-containing pre-tRNA^Tyr^ with immature 5′ leader and 3′ trailer sequences acquired 5-methylcytidine (m^5^C) in the variable loop, 1-methyladenosine (m^1^A) in the TΨC loop, and pseudouridines in the TΨC and anticodon loop. As soon as the 5′ and 3′ ends became matured, other new modifications were added, which include additional pseudouridylations and dihydrouridine to the D-loop [126,127]. The process of this modification is different in each mature tRNA species. Some modification enzymes are architecture-dependent enzymes that need a fully folded tRNA for activity, and others are architecture-independent enzymes that do not need a fully folded tRNA for activity [121]. Therefore, it is crucial to design an o-tRNA architecture to meet the structural requirements of its modifying enzymes. A proper understanding of these specifics will further facilitate the efficiency of incorporation.

Regardless of the approach to fine-tune o-tRNA and aaRS interaction, tRNA molecules are targets of a wide range of enzymes that have a direct impact on tRNA activity in the cell (their half-life, processing, cellular localization) and, consequently, affect the site-specific incorporation of amino acids [112,128]. It has been reported that long-term nutrient limitation leads to tRNA degradation, and the tRNA half-life may be decreased under conditions such as amino acid starvation, where a reduction of near-cognate tRNA pools might improve translation accuracy [129]. Oxidative stress also affects the half-life of tRNAs. A growing body of studies has shown that in eukaryotic and prokaryotic cells, cleavage of tRNAs due to oxidative stress leads to the formation of small RNA fragments that can repress translation initiation [125]. However, recent studies have provided increasing evidence that a considerable fraction of genes is more actively translated under oxidative stress in eukaryotic cells. For example, the hypermodification of tRNA^Leu(CAA)^ at the wobble position enhanced the translation of TTG codons after exposure to H_2_O_2_ and thus improved the protein expression of TTG-enriched genes [92].

### 3.2. Modulation of o-tRNA Transport across Cellular Barriers in Eukaryotic Expression Hosts as a Means to Improve GCE

The transport of tRNAs across cellular membranes to new locales within the cell offers a given tRNA the potential to be further modified depending on the modification enzymes present and the substrate structural landscape. When a tRNA is localized to a new compartment where it is recognized by a different set of enzymes, which is not usually a substrate, this may lead to the formation of hypomodified tRNA. There is also the possibility that the tRNA may acquire new modifications that it does not usually get. Since intracellular transport dynamics are linked to many processing events and certain modifications, the transport of o-tRNA across cellular barriers can be engineered to facilitate transport across cellular membranes and modification of o-tRNA to optimize the site-specific incorporation of ncAAs. The mature tRNA with the correct addition of CCA and a proper secondary and tertiary structure are bound by exportin-T or exportin-5. Exportin-T preferentially binds end-processed tRNAs in vitro, and it does not discriminate between tRNAs with or without introns, which is an essential feature of tRNA intracellular transport when the splicing machinery localizes to the cytoplasm [130,131,132]. In *S. cerevisiae*, Msn5 is the primary transporter for tRNAs, which have been retrogradely transported to the nucleus after splicing, and therefore binds to mature aminoacylated tRNA [133]. Los1p, the nuclear pore protein, binds tRNA in a Ran-GTP-depedent mechanism, and its degradation leads to nuclear accumulation of tRNAs [134,135]. Besides the aforementioned tRNA transporters, other proteins of moonlighting cellular functions have been shown to affect tRNA localization. As an example, *S. cerevisiae* Sol1p and Sol2p (whose genes are homologues to genes encoding 6-phosphogluconolactose in the pentose phosphate pathway) are involved in tRNA nuclear export, which accounts for their effects on tRNA-mediated nonsense suppression. Overproduction of Sol1p, Sol2p, and Msn5 in *S. cerevisiae* might prevent the accumulation of intron-containing orthogonal pre-tRNAs. Furthermore, the glycolytic enzyme glyceraldehyde 3-phosphate dehydrogenase (GAPDH) binds to tRNA and aids in the nuclear export of tRNA in a saturable and carrier-mediated process [136].

o-tRNA should be designed to enhance processing by improving transport across cellular membranes. When selecting the specific o-tRNA to be modified, it is essential to identify the intracellular localization of the modifying enzymes and understand whether splicing is a prerequisite for such modification. In *S. cerevisiae*, different enzymes are localized to the nucleus, cytoplasm, or mitochondria, where they participate in tRNA modification (for detailed information, check [128]). Hypomodification of o-tRNA could also affect the export potential of o-tRNA from the nucleus, and this may also limit the yield of synthesized non-natural proteins. The thiouridination of spliced tRNA in *S. cerevisiae* can occur either in the cytoplasm by Uba4, Ncs6, and Ncs2 [137] or in the mitochondria by Mtu1 [138]. Some other modification enzymes present in the cellular nucleus of *S. cerevisiae* include Trm1 (N^2^,N^2^-dimethylguanosine (m^2,2^G)) [139], Trm4 (5-methylcytidine (m^5^C_40_)) [113], Trm5 (1-methylguanosine (m^1^G_37_)) [120], and Pus1 (pseudouridine (Ψ_34_ and Ψ_36_)) [120], while some other enzymes, such as Pus3 (pseudouridine (Ψ_38_ and Ψ_39_)) [140], Pus8 (pseudouridine (Ψ_32_)) [141], Trm11 (N^2^-methylguanosine (m^2^G_10_)) [142], and Trm7 (5-carbamoylmethyl-2′-O-methyluridine (ncm^5^Um)) [143] are localized to the cytoplasm. Therefore, seeing how tRNA cellular localization affects the structure and stability of the designed o-tRNAs, it is reasonable to speculate that engineering of the o-tRNA transport system to prevent nuclear accumulation and facilitate proper intracellular localization is crucial for synthesizing non-natural proteins.

### 3.3. Modifications of the Target Protein Producer Organism to Allow an Efficient o-tRNA Processing

In the biological system, the role of RNA modification in post-transcriptional gene regulation, biogenesis, and the stability of RNA has been widely studied. Modifications of tRNA are widely used to enhance codon–anticodon interaction and maintain the anticodon loop structure. MODOMICS, a database of RNA modifications, encompasses over 170 different RNA modifications with up to 93 modifications specified in tRNAs [144,145], thus indicating that tRNAs have the highest density of post-transcriptional modifications among all other RNAs. Notably, certain regions on tRNA structures have been signified most sensitive to modification, including the region encompassing nucleotides of the anticodon loop and the tRNA core region. The overall effect of tRNA modification has been shown to optimize wobble interaction, fine-tuning of tRNA structure, contribution to thermophilic adaptation, and psychrophilic adaptation [146].

Recently published data by Crnković et al. [36] showed the effects of heterologous tRNA modifications on the production of proteins with incorporated ncAA O-phosphoserine (Sep) in *E. coli* cells. The insertion of Sep into a green fluorescent protein (GFP) in a library of *E. coli* strains carrying genes that were either deleted or overexpressed by single tRNA modifications was used to visualize different genes that affected o-tRNA activity. A positive correlation was observed between dimethylallyl transferase MiaA and pseudouridine synthase TruB activity, due to their overexpression, and the improved specificity of Sep incorporation. In the *iscS* mutant, which has no cysteine desulfurase activity, the yield of Sep-containing GFP increased by eight-fold. The enzyme has a major role in the pathway for iron–sulfur (Fe/S) cluster biosynthesis and tRNA modification in *E. coli*.

Epitranscriptomic studies on o-tRNAs are needed to fully harness the potentials of o-tRNA modifications in the incorporation of ncAA into proteins. Among the several modifications reported in tRNA, many have been observed to target the anticodon stem-loop, particularly base 34, which is involved in wobble base pairing, and base 37, which is 3′-adjacent to the anticodon [13,147]. Besides the canonical function of tRNA modifications in translation, several tRNA modifications seem to regulate the activity of metabolic networks of the host cells.

#### Improvement of Host Organism’s Cellular Fitness upon Alteration of Its Metabolism by Integrated o-tRNAs

**Figure 3:**
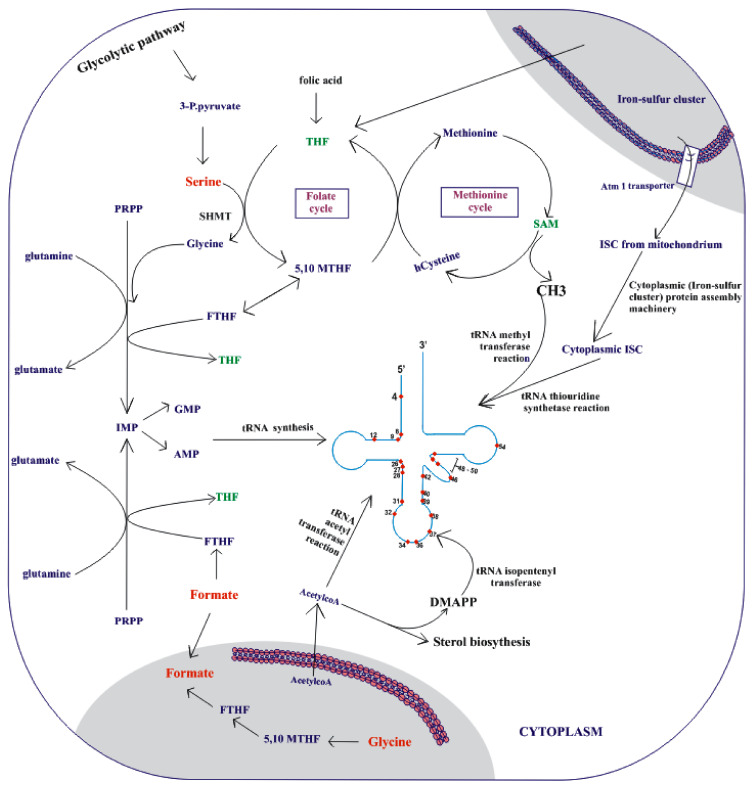
The metabolic pathways that provide precursors necessary for the tRNA modifying enzymes in eukaryotes. The mitochondrion metabolic activity plays a vital role in tRNA modification. The single-carbon metabolism in the mitochondrion maintains the appropriate cytoplasmic pool of tetrahydrofolate (THF). The folate cycle cross-talks with the methionine cycle in a highly dependent manner, thereby providing SAM, a methyl donor for tRNA methyltransferase reaction. The mitochondria also supply an iron–sulfur (Fe/S) cluster that is transported via the Atm1 transporter into the cytosol. The cytoplasmic iron–sulfur protein assembly machinery produces the cytoplasmic iron–sulfur cluster that is essential for tRNA thiolation. In addition, intermediary metabolic pathways in the mitochondria usually produce acetyl-CoA, which serves as a donor of acetyl moiety for the tRNA isopentenylation pathway and most acetyl transferase reactions, including tRNA acetylation. The glycolytic pathway serves as a major source of energy for tRNA synthesis. Overall, the modification hotspots of tRNA are also shown, including their positions. (SAM: S-adenosylmethionine; 3-P. pyruvate: 3-phosphohydroxy pyruvate; ISC: iron–sulfur cluster; 5,10 MTHF: *N*,*N*-methylene tetrahydrofolate; FTHF: 10-formyltetrahydrofolate; SHMT: serine hydroxymethyltransferase; PRPP: phosphoribosyl pyrophosphate; DMAPP: dimethylallylpyrophosphate).

Although there are several gaps in understanding of the whole cell biology, the connections between nutrient sensing, signaling pathways, and tRNA metabolism are increasingly well recognized. Several tRNA modification defects trigger *GCN4* signaling in the absence of amino acid starvation [148,149]. Translation itself is tightly bound to nutrient availability via tRNA conserved chemical modifications. The modifications consume metabolites produced by several pathways, among them are sulfur metabolism, S-adenosylmethionine (SAM), cysteine, and DMAPP (Figure 3). So far, the synchronization of translation with metabolism and amino-acid-sensing mechanisms and amino acid homeostasis has been shown to be reflected by the levels of uridine thiolation at the tRNA anticodon, which is growth dependent [150]. Therefore, at least in *S. cerevisiae*, under certain growth conditions, some types of tRNAs, such as tRNA^Lys^ (K), tRNA^Glu^ (E), and tRNA^Gln^ (Q), appear as growth controllers. tRNA thiolation facilitates wobble base pairing. This brings several implications to the GCE RS/o-tRNA-based systems when o-tRNAs are overproduced in a target host organism. For instance, if o-tRNA has uridine at 34 position and/or adenosine at position 37, the GCE performance might decrease in yeast factories if rich media has not been additionally supplemented by reduced sulfur equivalents, sulfur-containing amino acids. Available literature suggests that the excess of tRNA (in theory also o-tRNA), when its fraction remains unthiolated, shall be tuned and modified to correctly reflect the nutritional conditions and intracellular sulfur amino acid availability under favorable growth conditions [150]. Some revealed data emphasize that tRNA levels reciprocally regulate amino acid and carbohydrate metabolism to help achieve metabolic homeostasis.

Lack of 2-thiolation of uridine residues at the U_34_ position of tRNA^Glu^, tRNA^Gln^, and tRNA^Lys^ (s^2^U_34_), which affect the efficiency of wobble base codon–anticodon pairing [151,152], has been shown to directly promote alteration of cellular metabolism, phosphate homeostasis, and upregulation of storage carbohydrates’ de novo synthesis [106]. The tRNA maturation pathway is saturated by the increased amounts of primary transcripts in cells lacking Maf1, the regulator of RNAP III. A study by Szatkowska et al. [153] provides evidence that increased activity of RNAP III in MAF1-deficient cells, with a reprogrammed tRNA pool [154], correlates with proteome reprograming and broad changes in several metabolic processes, including glycolysis, amino acids, and trehalose biosynthesis, glycine catabolic process, and purine ribonucleotide synthesis. In an *maf1Δ* background, the so far identified limited modification of tRNA involves Trm1 methyltransferase activity catalyzing m^2^_2_G26 tRNA modification, which plays a crucial role in the reduction of the activity of a tRNA_UCA_^Ser^ suppressor and accounts for antisuppression [155]. This observation further suggests that in GCE application, the excess of specific o-tRNAs (if targets to modifications due to lack of full orthogonality) will change tRNA pool composition, causing a burden to the host metabolic networks. This is likely due to the adaptive cellular response, leading to the increased demand for chemical compounds required for tRNA modification and activities of modifying enzymes. Not only in the anticodon loop, tRNA modifications often require multiple enzymes and µM concentrations of many substrates, metabolic intermediates. In the end, improving of the host cell capacity for o-tRNA complex modifications may reduce the adverse effects of o-tRNA overdose (increasing hypomodified tRNA pool) on ncAA insertion manifested with growth inhibition.

As an example [103], cells expressing *Mj* tRNA_AUG_^Opt^ for sense codon reassignment, which is modified to tRNA_IUG_^Opt^, adversely affect the cell growth rates. Prevention of inosine 34 modification of Mj tRNA_AUG_^Opt^ by mutations at positions 32, 37, and 38 in anticodon loop variants increases relative maximal optical density from 84% to 90% of the control no sense reassignment systems. It is however not clear from this study whether editing of adenine to inosine in o-tRNA compromises a cell’s health or the other modifications present at positions 32, 37, and 38 are their direct cause. For instance, the mutation A37 to G37 in clone B5 from the library would prevent *Mj*tRNA_AUG_^Opt^ 2-methylthio-N^6^-isopentenyladenosine (ms^2^i^6^A) modification, whose synthesis requires a whole cascade of enzymatic reactions.

In theory, if there is a competition between o-tRNAs and tRNA^Glu^, tRNA^Gln^, and tRNA^Lys^ for any of those enzymes involved in thiolation in yeast, a substantial decrease in overall translational activity would be expected. Genes highly enriched for all three (K, E, Q) codons are substantially over-represented in rRNA processing, ribosomal subunit biogenesis, and other translation/growth-specific biological process [150]. If the production of proteins with enzymatic activities is adversely affected, such as GAPDH, that would directly affect the tRNA concentration in the cytoplasm, see Section 3.2.

### 3.4. Upregulation of Metabolic Precursors for o-tRNA Modifications

The functions of tRNA molecules can be modulated by modifications that play different roles depending on the specific modification and position in the tRNA molecule. The modification level depends on the substrate availability of the precursor metabolites that participate in the modification reaction (Figure 3). For example, the discovery that the ms^2^i^6^A37 synthesis protein MiaB was an iron–sulfur (Fe/S) cluster-dependent enzyme explained how sulfur and iron levels could regulate this modification [156]. *E. coli* cells grown in iron-deficient media contained tRNAs harboring lower levels of ms^2^i^6^ A37, thereby lowering the efficiency of polynucleotide synthesis in vitro and resulting in an increase in the synthesis of amino acid operons [157]. Similarly, a recent report demonstrated the role of the tRNA modification m^1^G37 in Mg^2+^ homeostasis in *Salmonella enterica* [158]. A low level of Mg^2+^ increased the transcription of the downstream Mg^2+^ transporter gene *mgtA*, and a mutated version of TrmD decreased the levels of m^1^G37-modified tRNAs, resulting in an increased expression of *mgtA.* The presence of bound Mg^2+^ ions is critical for establishing the compact L-form of tRNA [159]. To advance the activity of o-tRNA in the synthesis of non-natural proteins, we highlight the key tRNA modification enzymes that play important roles in the improvement of translational fidelity (Table 2) and the different modification pathways involved (Figure 3).

#### 3.4.1. Methylation as a Target

Methylation is one of the post-transcriptional modifications that can fine-tune tRNA function in many cellular processes. In fact, aberrant modification of tRNA can contribute to various metabolic disorders in metazoans and metabolic changes in single-cell organisms [160,161]. S-adenosyl-L-methionine (SAM) is a vital methyl donor for tRNA methylation reaction. This reaction is catalyzed by tRNA methyltransferases (Figure 3) occurring usually as 2^1^-O-ribose methylation in all the canonical RNA nucleotides [162]. In *E. coli*, three methylation sites have been identified, including the 2^1^-O-methylation of guanine at position 18 (Gm18) in the D-loop and 2^1^-O-methylation of cytosine and uridine nucleotides at positions 32 and 34 in the anticodon loop [135] (Table 2).

Notably, these positions are not completely conserved in all bacteria. For instance, certain species of *Mycoplasma* may lack Gm18, but contain methylated uridine at position 34 of the anticodon loop [107]. Although the positions of methylation may differ significantly for most higher organisms, Gm18 is highly conserved in *S. cerevisiae* [163]. In a report by Bjork et al., modification of tRNA by nucleoside 1-methylguanosine (m^1^G) prevented translational frameshifting, while lack of m^1^G in tRNA reduced the growth rate by 40% at 41 °C in *Salmonella typhimurium* [164]. Their study concluded that the conservation of m^1^G37 in tRNA_CUN_^Leu^, tRNA_CGN_^Arg^, and tRNA_CCN_^Pro^ during evolution prevents frameshifting regardless of the organisms involved. In addition, the methylation of tRNA was revealed to modulate immune stimulation in a particular study where the 2^1^-O-methylation of guanine in bacteria suppressed the activation of TLR7/TLR8 in human immune cells [165]. Studies have shown that such modifications contribute to conformational rigidity and translational fidelity [166,167]. Coexpression of archaeal Trm5 methyltransferase, which catalyzes the methylation of tRNA^Sep^ G37 in *E. coli*, significantly improved tRNA^Sep^ orthogonality [36]. An N^2^,N^2^-dimethylguanosine (m^2^_2_G)-abundant modified nucleotide is present in tRNAs from archaea and eukaryotes, but not in eubacterial tRNAs [168]. In eukaryotic tRNAs, m^2^_2_G is only found at position 26. In general, the findings from these studies demonstrate the importance of methylation in the maintenance of cell physiology.

#### 3.4.2. Thiolation as a Target

As previously ascertained, post-transcriptional modification of tRNA is critical to the structure and function of tRNAs. In many organisms, the wobble position of tRNA is highly predisposed to modification, and when the modifications are present, the decryption of genetic codes is enhanced [169]. For instance, the thiomodification 5-methyl-2-thiouridine xm^5^s^2^U or s^2^U thiolation alone of tRNA^Lys^, tRNA^Gln^, and tRNA^Glu^ at wobble position 34 was reported to be crucial to ensuring accurate deciphering of the genetic code due to increased ribosomal A-site binding, reduced ribosomal pausing at critical codons, or reduced ribosomal frameshifting as well as stabilization of the tRNA structure [115,149,170,171,172]. The tRNA modification xm^5^s^2^U is universally conserved in bacteria (mnm^5^s^2^U), mitochondria (τm^5^s^2^U), and eukaryotes (mcm^5^s^2^U). The function of U34 2-thiolation in promoting the efficient translation of cognate codons is conserved from yeast to metazoans. Yeast mutants defective in mcm^5^/s^2^U modification were identified to exhibit mitochondrial translation defects at elevated temperatures, which result in respiration deficiency [173].

Importantly, the process of thiolation requires enzymatic reactions, cofactors, and metabolic activity of mitochondria [107]. Although this process of tRNA thiolation occurs in the cytoplasm, sulfur required by modifying enzymes for tRNA thiolation is provided by the mitochondria. The Atm1 transporter is engaged in the process of sulfur export from mitochondria to the cytosol [174,175]. Specifically, mitochondria possess iron–sulfur (Fe/S) cluster machinery that provides the iron–sulfur intermediate necessary for the cytoplasmic iron–sulfur protein assembly machinery [175]. This cytoplasmic iron–sulfur cluster is significant for thiolation reaction (Figure 3).

In thermophiles, the thiolation of methyluridine at position 54 is universally conserved. It involves a nonredox replacement of C2-uridine carbonyl oxygen by sulfur, and it is catalyzed by tRNA thiouridine synthetase. Worthy of note is the importance of the Fe/S cluster and ATP in the catalytic efficiency of this enzyme [176]. While the Fe/S cluster is particularly important in thermophilic bacteria and hyperthermophilic archaea, there is a dearth of information on its role in the thiolation of eukaryotic tRNAs [177,178]. However, as one of the major types of modification, thiolation produces many effects on tRNAs and the host cell. These include their role in biogenesis, metabolism, and host cell stability at the cellular level. There are phenotypic consequences associated with defects in the ubiquitin-related modifier-1 (URM1)-dependent tRNA_UUU_^Lys^, tRNA_UUG_^Gln^, and tRNA_UUC_^Glu^ thiomodifications in *S. cerevisiae*, such as activation of multiple stress-responsive genes [179], and conditional advantages for cell survival, such as conferring resistance to endoplasmic reticulum stress [180].

#### 3.4.3. Acetylation as a Target

A growing body of evidence has also shown the importance of acetylation in tRNA function. This modification can also occur at the anticodon loop of tRNAs. Through the process of acetylation, the ataR–ataT complex, which was reported as a novel toxin–antitoxin system from i *E. coli*, was used to modify Met-tRNA^fMet^. The transfer of the acetyl group from acetyl-CoA led to translation inhibition and the formation of dormant persisters [181]. In addition, the acetylation of tRNA is also possible without the involvement of acetyl-CoA. In *Bacillus subtilis*, it was uncovered that the formation of N^4^-acetylcytidine of bacterial tRNA^Met^ at wobble position 34 does not require acetyl-CoA. Taniguchi et al. identified the TmcAL gene [182]. They showed that this gene caused the activation of acetate ion, leading to the formation of acetyladenylate and ultimately the catalysis of the reaction that forms N^4^-acetylcytidine 34. tRNA acetylation has also been reported to play essential roles in the toxin–antitoxin system of infectious agents and key players in the underlining antibiotic resistance mechanisms. For instance, the formation of antibiotic insensitive persisters in the *Salmonella* population was correlated to the presence of a toxin called TacT. This toxin is an acetyltransferase enzyme that hampers the translational process by transferring acetyl groups from acetyl-CoA to primary amine moiety on charged tRNAs, thus leading to the formation of nongrowing antibiotic recalcitrant cells. However, these cells can resume growth through the peptidyl-tRNA hydrolase-mediated detoxification mechanism [183]. This mechanism may be associated with the recidivism of infections. Overall, it is not uninformative to speculate that tRNA acetylation is also critical to the decryption of codes because tRNA acetylation also takes place at the wobble position.

#### 3.4.4. Isopentenylation as a Target

Modifications of the base at position 37 mostly include large additions to adenosines, such as N^6^-isopentenyl adenosine (i^6^A) and 2-methyl-thio i^6^A (ms^2^i^6^A). Available data reveal that i^6^A and its derivates are exclusively found at position 37 (Table 2). The i^6^A modifications are present in bacteria and eukaryotes but absent in archaea. Reports have shown that the A–U base pairs on the first codon position (which is the base 36 of tRNA) requires a base 37 that has been modified in order to ensure the Watson–Crick base pair’s stability by base stacking [122]. Additionally, this modification prevents the unwanted hydrogen bonding between U33 and A37, thereby ensuring the anticodon loop’s stability [122,184]. In *E. coli*, the biochemical pathway that leads to the formation of i^6^A or ms^2^i^6^A (2-methyl-thio i^6^A) was initially discovered with *mia*A mutants lacking ms^2^i^6^A in tRNA^Trp^. Thereafter, *mia*B was recognized as the required gene for the synthesis of ms^2^i^6^A, and *mia*E was found to catalyze ms^2^io^6^A in *S. typhimurium* [185]. Isopentenyladenosine is then subsequently modified to ms^2^i^6^A by MiaB and can be hydroxylated by MiaE to ms^2^io^6^A in *S. typhimurium*. MiaB belongs to the family of SAM enzymes that play key roles in tRNA modification. In a report by Gefter and Russell [186], the results obtained from ribosomal binding assays showed that *E. coli* tRNAs without natural i^6^A or ms^2^i^6^A had reduced binding affinity to their cognate codons. Likewise, the lack of isopentenylation of tRNA^Trp^ in a mutant that was defective in the attenuation of *miaA* also led to the impairment of the function of the nonisopentenylated tRNA^Trp^ [187]. The absence of isopentenylated tRNA also caused a marginal decrease in the growth of *E. coli* in a full culture media. *S. typhimurium mia*A mutants also revealed a reduction in the growth rate and protein production on several substrates [188,189]. In *S. cerevisiae*, *mod*5-1 mutation showed decreased levels of i^6^A, thereby reducing the efficiency of a tRNA_UAA_^Tyr^ suppressor [122]. *maf1-1* mutation decreases the efficiency of tRNA suppression. This suggests that proper modification of tRNA through isopentenylation is highly essential for the growth of organisms on a particular growth substrate and the corresponding protein synthesis.

#### 3.4.5. Adenosine-to-Inosine Editing as a Target

The post-transcriptional process involving base deamination is a prominent editing mechanism that has an impact on tRNA’s overall structure and function [190]. One of the most common types of deamination in archaea, bacteria, and eukaryotes is the conversion of adenosine (A) to inosine (I). Both eukaryotes and prokaryotes utilize adenosine-to-inosine editing to expand each tRNA’s decoding efficiency and limit the number of tRNA species needed for codon–anticodon identification [191]. Adenosine deaminase, a two-subunit (Tad2 and Tad3) enzyme, catalyzes the hydrolytic deamination of adenosine to inosine at wobble position 34 of tRNAs in *S. cerevisiae*. [192]. I_34_-modified tRNAs can wobble-pair with A, C, or U at the third position but not G, thereby increasing the number of codons that genetically encode A_34_ tRNAs, which can recognize by threefold [193]. In a bacterial cell, the tRNAs modified with inosine at position 34 are tRNA_ACG_^Arg^ and tRNA_AAG_^Leu^. In eukaryotes, the tRNAs modified at position 34 are tRNA_AGU_^Thr^, tRNA_AGC_^Ala^, tRNA_AGG_^Pro^, tRNA_AGA_^Ser^, tRNA_AAG_^Leu^, tRNA_AAU_^Ile^, tRNA_AAC_^Val^, and tRNA_ACG_^Arg^, and in position 37, it is tRNA_AGC_^Ala^ [192,193]. In bacteria, adenosine deaminase A (TadA), which is essential for tRNA maturation, modifies tRNA_ACG_^Arg^ to tRNA_ICG_^Arg^ [118]. tRNA^Arg^ is identified up till now as the only A_34_-containing tRNA known to be deaminated to I_34_ in bacteria, and this gives an explanation to why bacterial arginine codons are preferentially translated by tRNAs initially transcribed with A_34_ [194]. *Mj*tRNA_AUG_^Opt^ has been identified as a substrate for A to I deaminase TadA, resulting in tRNA_IUG_^Opt^, which causes lack of discrimination between two histidine codons, CAU and CAC [91]. A directed evolution of the *Mj*tRNA_AUG_^Opt^ anticodon loop prevents adenosine 34 editing to inosine. tRNA_AUG_^Opt^ derivatives eliminate o-tRNA recognition by TadA and improves the reassignment efficiency of the sense CAU codon.

### 3.5. tRNA Modification Tunable Transcripts

To further explore the roles of tRNA modifications in the synthesis of non-natural proteins in the GCE system, we review the roles of modification tunable transcripts (MoTTs) in the translational regulation of critical stress response proteins and biased codons recoding for ncAA incorporation. MoTTs are transcripts whose translation is regulated by changes in wobble base modification. The reprograming of tRNAs through the chemical modifications of their anticodon interface is involved in the selective translation of proteins encoded by degenerate codons. This principle is used to incorporate Sec in ciliates during translation as an important regulatory component of stress response proteins against oxidative stress. A strong correlation exists between wobble base modifications and the encoding of selenocysteine [195]. The internal UGA stop codon in mRNA is used to encode Sec when tRNA^Sec^ is modified at uridine wobble base position 34. The presence of methoxycarbonylmethyluridine (mcm^5^U) and 5-methoxycarbonylmethyl-2′-O-methyluridine (mcm^5^Um) modifications improves the anticodon–codon interactions required to decode selenocysteine-containing proteins [196]. Similarly, the modification of C to m^5^C in *S. cerevisiae* by Trm4 at the wobble position of tRNA^Leu^ increases when exposed to H_2_O_2_, which consequently stimulates the translation of mRNAs (MoTTs) derived from 38 genes in yeast in which 90% or more of the leucines are encoded by UUG [92].

In budding yeast, 9 out of the 24 modified ribonucleosides are present at wobble position 34, and the majority of these wobble modifications occur at uridine [197]. This increases the flexibility of uridine in degenerative codon usage in response to the stimulating agents, such as oxidative stress, DNA damage, and alkylating agents. Another important feature of MoTTs by wobble modifications in the GCE system is their frequent tRNA specificity, which suggests their importance in the site-specific incorporation of ncAAs. For example, tRNA methyltransferase 4 (Trm4) catalyzes the formation of m^5^C at position 48 in 34 tRNA species, but only tRNA^Leu^ has m^5^C at the wobble position [144]. Taken together, tRNA modification reprogramming in relation to MoTTs serves as a distinct blueprint for translational regulation of non-natural protein synthesis by signaling “on” or “off” of specific transcripts. This method can be used as gene therapy in treating genetic disorders.

## 4. Applications of Genetic Code Expansion

### 4.1. Studies on Post-Translational Modifications (PTMs) of Proteins by Incorporation of ncAA

Codon expansion in protein synthesis explores diverse applications in post-translational modification (PTM) functions. For instance, enzymology investigations including lysine 2-hydroxyisobutyrylation, phosphorylation of serine, tyrosine, threonine, acetylation, and crotonylation of lysine [198,199,200,201,202] and mapping of protein interactions including interactions involving protein–protein [203] and protein–DNA [204] can be enhanced through codon expansion, as well as the structure and functions of proteins. Importantly, this can be achieved with the aid of ncAAs incorporated with fluorescent groups’ side chain, which is photoactive, and such photoactive side chain includes p-benzoyl-l-phenylalanine [66]. The principle involves the use of photo-crosslinking amino acids that can be chemically cleaved following crosslinking and the incorporation of ncAAs with fluorescent groups, which can be identified with the aid of a mass spectrophotometer [203]. It has also been shown that phosphoramide can be deprotected in proteins that are tolerant to acidic deprotection (such as ubiquitin) by encoding a phosphoramide precursor to phosphotyrosine [205]. Another study shows that the deletion of phosphatase after mutating EF-Tu prevents the hydrolysis of amino acids, thereby allowing a mixture of amino acids to be incorporated, including phosphotyrosine, at a low protein yield [206]. Phosphoserine (pSer) has also been encoded using a SepRS-tRNA_CUA_ pair during the incorporation of cysteine into proteins in a particular group of methanogens, including *M. jannaschii*, *Methanothermobacter thermautotrophicus*, *Methanococcus maripaludis*, *Methanococcoides burtonii*, *Methanospirillum hungatei*, *Archaeoglobus fulgidus*, and *M. kandleri* [200]. Further studies achieved efficient pSer incorporation when *E. coli* was used as the host organism [206,207]. EF-Tu was engineered to accommodate pSer and prevented its cleavage, while the SepRS-tRNA_CUA_ pair was evolved to efficiently function as an amber suppressor. Rogerson et al. proposed rewiring the cellular metabolism of *E. coli* to encode a nonhydrolyzable phosphate analogue of pSer and feeding the cells with the analogue. The application of a genetic code extension in the post-translational modification of proteins and their nonhydrolyzable analogues opened new frontiers that allowed exploration to specifically direct the in vivo consequences of PTM protein phosphorylation at specific residues [208]. This GCE approach was demonstrated to be useful to investigate the effects of histone acetylation on a chromatin structure in vitro and to study the epigenetic regulation of a gene expression in genetically directed histone acetylation in chromatin in vivo in cell lines [202,209,210].

### 4.2. Improvement of Protein Stability for In Vitro and In Vivo Studies

With the application of codon expansion, protein stability can be enhanced to produce proteins with in vivo and in vitro thermal stability [211]. For instance, ncAAs incorporating long side-chain thiols, such as p-isothiocyanate phenylalanine or p-fluorophenylalanine, into β-lactamase results in disulfide bond formation with characteristically better thermal stability at 37 °C than the G-U wobble base pair of the natural anticodon sequence (GAA) encoding phenylalanine [104]. A novel ncAA (pNCSF) was reported by Xuan et al. to contain an aryl isothiocyanate side chain that formed a stable intramolecular thiourea crosslink with amines under mild conditions and was genetically encoded into proteins in *E. coli.* pNCSF was incorporated into myoglobin to replace a native salt bridge by forming intramolecular crosslinks with a proximal lysine residue, which improved the protein’s thermal stability [212]. Other amino acids that form intramolecular crosslinks within protein have been incorporated into proteins using this method. Examples of such amino acids include boronate groups [213], alkynes [214], amino acids with reactive halides (e.g., haloalkanes and α-haloketones) [215], asides [216], photoreactive side chains (e.g., aryl asides and diazirines) [66], and Michael acceptors (e.g., sulfonamide groups and α,β-unsaturated carboxylamide) [217].

### 4.3. Engineering of Proteins with Enhanced Functions

Codon expansion in protein synthesis technique prevents the reaction of residues with the immobilizing agent during biocatalyst preparation, as in the case of the inclusion of a single ncAA used for immobilizing reaction, into the desired sites (sites that bring about the reduced effect of the original catalytic properties of the enzyme). This helps in the prevention of multiple residues that may react with the immobilizing agent during the preparation of biocatalysts [218]. Similarly, site-specific incorporation of ncAA into an immobilized enzyme can be used to build enzyme-based detecting devices. For instance, a magnetic bead system that uses N-methyltryptophan oxidase can detect a marker for poisoning by abrin (a natural lethal biotoxin) [219]. Recently published data provide evidence on a facile strategic method for the site-specific directional immobilization of enzymes on direct electron transfer electrodes [220]. In addition, 4-Azido-L-phenylalanine (AzF) was incorporated into specific sites of a small laccase from *Streptomyces coelicolor* (SLAC) on a multiwalled carbon nanotube electrode using copper-free click chemistry-mediated immobilization. This approach produced a remarkably increased direct electron transfer efficiency and a stable current that lasted for about 8 days of solution-phase incubation at ambient temperature [220,221]. The incorporation of ncAA into fluorescent proteins has also been shown to further improve their function as highly efficient sensing biodevices. The cotranslational incorporation of p-boronophenylalanine into fluorescent proteins (FPs) using a fluorescent protein biosensor has been used for the in vivo and in vitro detection of hydrogen peroxide, and the incorporation of p-azidophenylalanine (pAzF) into fluorescent proteins enhances the sensing and detection of hydrogen sulfide (a vital gastrotransmitter) [222,223]. The use of ncAAs to improve the activity and selectivity of existing enzymes enhances the versatility of enzyme catalysis, thus providing rapid access to novel enzymes with new catalytic activities. For example, the introduction of formyl glycine into sulfatases, 4-methylideneimidazole-5-one (MIO) into lyases, and p-aminophenylalanine into a lactococcal multidrug resistance regulator improved their functionalities [224]. The incorporation of *O*-*tert*-butyltyrosine and *p*-phenylphenylalanine at position 222 enhanced the enantioselectivity of diketoreductase, an industrially relevant enzyme that is capable of stereoselective reduction of ketones to chiral alcohols [225]. Other applications of genetically encoded ncAAs in biocatalysis including novel metal-binding sites and ancillary functions were reviewed by Drienovská and Roelfes [226].

In a study, the evolutionary advantage of GCE in the development of β-lactamase with enhanced catalytic activity was examined [227]. This was performed by generating an extensive library of β-lactamase variants with novel ncAAs, which were substituted at each site randomly throughout β-lactamase (TEM-1)**.** The β-lactamase variants with enhanced catalytic activity were isolated using a growth-based selection from this library of mutants containing single amber codon mutations. Similarly, in another study, TEM-1 β-lactamase was re-engineered to be addicted to ncAA (3-nitro-L-tyrosine or 3-iodo-l-tyrosine) and was maintained for hundreds of generations without escape [228] through computational design [8,229]. Therefore, by incorporating genetically encoded ncAAs into a natural genetic code, it is possible to produce beneficial evolutionary organisms with unique features by the directed evolution of proteins and the whole organism.

### 4.4. Construction of Biosynthesized Therapeutic Peptides

An extensive cyclic peptide library was constructed by Niu et al. using codon expansion in conjunction with split intein catalyzed ligation of proteins and peptides (SICLOPPS) that was successively coupled to advance an HIV protease inhibitor [230]. This was also used in therapeutic peptide biosynthesis when ncAA was incorporated to expand the chemistry and structure repertoire of natural products [231]. For instance, incorporation of ncAAs, such as p-acetylphenylalanine (pAcF), p-azidophenylalanine (pAzF), and Prock at distinct sites on nisin using an either *M. jannaschii* tRNA^Tyr^/TyrRS or *M. barkeri* tRNA^Pyl^/PylRS system in *Escherichia coli*, was used to expand the building blocks of nisin [231]. The incorporation of hydroxy acids into the precursor peptides of two lantibiotics, lactin 481 and nukacin ISK-1, was used to cleave a leader peptide by simple hydrolysis in *E. coli* [232]. The structural diversity of peptide antibiotics can be expanded by the incorporation of isostructural and orthogonal ncAAs into the lasso peptide capistruin. This was achieved with the use of supplementation-based incorporation (SCI) and stop-codon suppression (SCS) methods [233,234]. Four ncAAs were used to replace natural amino acids (NAA) at four sites in lasso microcin J25 (mccJ25) with the use of the amber suppression method [234]. The biosynthesis of lasso peptide enzymes by the incorporation of ncAAs into ribosomally synthesized and post-translationally modified peptides (RiPPs) produced antibiotics with greater efficiencies than conventional antibiotics. Other studies have also shown that GCE was successfully employed to introduce ncAAs into macrocyclic peptides [133,136], thiopeptides [235], lanthipeptides [232,236], and natural product cinnamycin [237].

### 4.5. Development of Bioconjugates for Chemo Drug Synthesis

**Figure 4:**
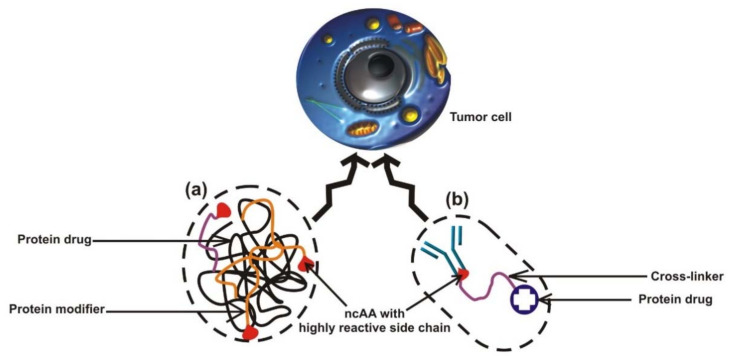
Modifications of protein-based drugs to target tumor cells. (**a**) The application of protein or chemo drug in tumor therapy using ncAA-mediated chemical alteration to improve the pharmacokinetic properties of the drugs to targeted tumor cells. A protein modifier (orange thread) can be used for the controlled drug delivery of a protein drug (black woven thread), thereby enhancing the drug action against tumor cell. All red droplets represent ncAA with a highly reactive side chain. (**b**) Schematic representation of bioconjugate used for the selective delivery of a drug of interest to cancerous cells using a drug–ncAA–antibody complex. The protein drug is conjugated to the reactive side chain of the ncAA by a cross-linker.

The codon expansion method entails the site-specific modification features, which makes it a powerful tool for improving and developing pharmaceutical proteins [231]. Genetic code expansion has widely been applied in chemo drugs and protein drug synthesis via incorporation of ncAAs with various moieties at one or more specific sites, including alkene, azide, and alkyne and ketone groups [238,239]. The pharmacological properties of rituximab, a drug used to treat autoimmune diseases and cancer, can be improved with the incorporation of ncAA. The conjugation of the anti-CD20 antibody rituximab with DIBO-DOTA (bifunctional linker) via Nε-2-azideoethyloxycarbonyl-L-lysine (NAEK) incorporation preceding radiolabeling is an important aspect in radioimmunotherapy [240]. When ncAAs are cotranslationally incorporated into the recombinant antibody, a bioconjugate is formed with the antibody and protein drug (Figure 4). This complex is then transported to the cancerous cells carrying the biomarker, which the antibody can detect. The cotranslational incorporation of ncAAs into a protein drug and modification with chemical modifiers improve the pharmacokinetic characteristics of the protein drug [241].

During the bioconjugation of chemo drugs and protein drug synthesis (targeted destruction of tumor cells), the incorporation of ncAAs contributes to the modern design of highly potent and promising anticancer drug delivery systems for targeted tumor therapy. For instance, incorporation of p-acetylphenylalanine (pAcF) into an anti-Her2 (Herceptin) antibody Fab, and conjugation to an auristatin derivative using oxime linkage, enhanced the in vitro cytotoxic effect of anti-Her2 against Her2 (Herceptin) positive tumor cells in xenograft typical rodents [242]. The therapeutic function of chemo drugs is enhanced by directing the capabilities of antibodies specific to the well-defined biomarkers on cancerous cells, thereby guiding the chemo drugs to their specific sites of action [231]. The incorporation of ncAAs makes the preparation of a bioconjugate simple and facilitates the homogenous preparation of an antibody–drug conjugate with good control of the conjugation site [243]. In a report of Lieser et al. [244], the construction of an effective anticancer drug delivery system for targeted tumor therapy was facilitated when therapeutic proteins were conjugated to an EGFR (epidermal growth factor receptor) ligand through an incorporated ncAA. Further enhancement of targeted protein delivery was achieved when the number of EGFR ligands was changed relative to specific proteins and when these ligands were clustered to increase the rate of ligand–receptor interactions. The strategy of an antibody–drug conjugate (ADC) by the incorporation of pAcF through genetic code expansion has also been utilized in the synthesis of chemically defined CXCR4-auristatin ADC and anti-CD11a IgG conjugated with a liver X receptor (LXR) agonist or phosphodiesterase 4 (PDE4) [245,246,247]. This technique has successfully been applied in developing immunosuppressive drugs when dasatinib, a drug for treating leukemia, was site-specifically conjugated with the humanized antibody HLCX, which specifically delivered an Lck inhibitor to human T lymphocytes [229]. The GCE strategy is universal; therefore, it may prove its usefulness to enhance binding specificity to various antigens by the single-domain V_NARs_ antibodies, which show superior penetration in tissue and solid tumors that are inaccessible to conventional big-size IgGs [248,249].

### 4.6. Immunotherapeutic of Bispecific Monoclonal Antibodies (BsMAb)

Bispecific monoclonal antibodies incorporate two or more antigen-recognizing elements into a unit, with the capability of binding to more than one target (epitopes). The approach basically relies on the engineering of numerous antigens binding domains into a unit molecule. Bispecific monoclonal antibodies are produced via chemical conjugations with the aid of residues, including cysteine or lysine embedded in the antibody. In some studies, an anti-CD3/anti-Her2 bispecific antibody was synthesized through copper-free click chemistry, occurring by encoding pAcF into anti-Her2 anti-CD3 Fabs, and then conjugation to the ethylene glycol linkers through an azide group on one end and alkoxy-amine on the other end [250,251]. This strategy is applicable in the synthesis of most of the bispecific antibodies. For instance, the synthesis of αCLL1-αCD3, according to Lu et al. [84], is a potential tool for an effective cure to acute myeloid leukemia as well as tumor in a xenograft model [252]. A variety of chemical reactions have been harnessed to advance new bispecific antibodies, such as in the development of anti-BCMA and anti-CS1 bispecific antibodies, which involve the reaction between tetrazine and bicyclononyne. The produced BiFab-BCMA effectively triggers T-cells and enhances prompt tumor regression [253]. Similarly, therapeutic bispecific targets have also been developed. They are made of a minute molecule called 2-[3-(1, 3-dicarboxy propyl)-ureido] pentanedioic acid, which selectively targets a prostate-specific membrane antigen (an antigen associated with tumor). When 2-[3-(1, 3-dicarboxy propyl)-ureido] pentanedioic acid was conjugated to pAcF and site-specifically expressed on anti-CD3 Fab, the EC_50_ of the conjugate was reduced, and this was used for the treatment of tumor cells in xenograft mouse models [254]. Kularatne et al. and Lyu et al. used the same technique to synthesize an anti-CD3 Fab-folate conjugate and a αGCN4-Fab conjugate, respectively [255,256].

### 4.7. Engineered Therapeutic Vaccines

**Figure 5:**
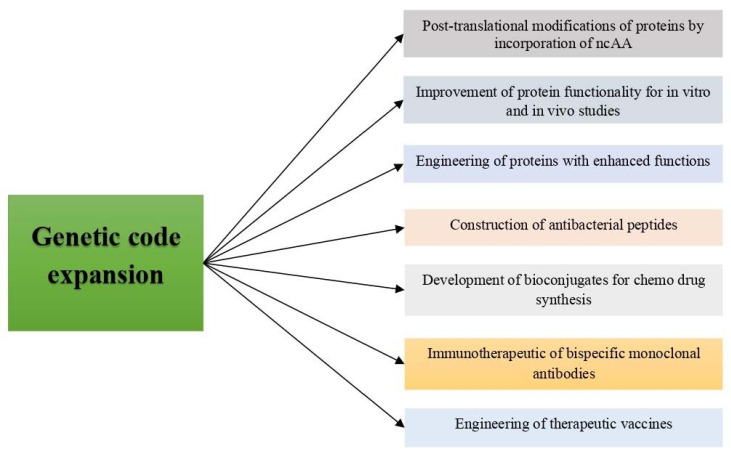
Applications of genetic code expansion.

The vaccination of patients is becoming a difficult challenge in health-care delivery due to weak immunogenicity and the development of self-immunotolerance by patients. In a bid to overcome these challenges, the potentials of GCE have been greatly harnessed. In previous studies, immunological self-tolerance has been broken with the use of various strategies, such as chemical derivatization of self-antigens [257] and adjuvants and the incorporation of foreign immunodominant T-helper (TH) epitopes into chimeric antigens [258,259] and DNA vaccines [260]. Codon expansion has been exploited to site-specifically introduce immunogenic ncAAs into autologous proteins and induce immune responses to self-tolerated epitopes in order to prevent the cross-reaction that has occurred between autologous tumor cells modified with dinitrophenyl and unmodified native tumor cell antigens [231]. Grunewald et al. reported that the site-specific incorporation of p-nitrophenylalanine (pNO_2_Phe) to murine tumor necrosis factor-α (mTNF-α) productively spawned a constant high-titer antibody response to parental and antigenically distinct mTNF-α variants, respectively, in the absence of any strong adjuvants [261]. Similarly, the incorporation of two related post-translational modifications, such as sulfotyrosine (SO_3_Tyr) and 3-nitrotyrosine (3NO_2_Tyr) of residues in mTNF-α and EGF, revealed that post-translational modifications show a significant and promising role in autoimmune disorders [262]. This technique has also been applied to a self-protein that is not related to immune function, murine retinol-binding protein 4 (RBP4) [263]. Overall, beyond the general applications of GCE systems highlighted in this review (Figure 5), advancing incorporation efficiency by tRNA modifications will create more opportunities to develop next-generation biotherapeutics. 

## 5. Conclusions

The application of a modified genetic code is gaining more relevance in a range of biotechnological, industrial, and clinical innovations. The application has already been found very useful in basic research. If adopted in the industry, it would offer GCE engineered non-natural protein catalysts as a new, cleaner, and safer alternative to the pharmaceutical manufacturing process currently based on chemical synthesis. The production of non-natural proteins with novel functional and structural properties can be improved by the optimization of the orthogonal translational system, engineering of orthogonal ribosomes, metabolic engineering of the host cells, and eventual evolvement of genomically recoded organisms. This review discussed specific knowledge on cellular factors affecting the specificity, the efficiency of ncAA incorporation into proteins, and the yield of non-natural protein synthesis. We highlighted that the optimization of a codon–anticodon interaction via tRNA modifications is tantamount to the maintenance of proteome integrity and the cellular metabolic homeostasis of the host organism, the protein producer. Discoveries of the new regulatory aspects of tRNAome undoubtedly will further facilitate the engineering of GCE translational systems, with o-tRNAs as their essential components. Future directions in the analysis of bacterial and eukaryotic cell metabolism and the integration of noncoding tDNA gene expression and activity of metabolic networks involved in a plethora of tRNA modification should be geared towards designing an optimal cellular environment to produce biotherapeutics and biocatalysts in metabolically engineered organisms. As the field of study develops due to its broadly recognized importance, we are optimistic that new applications on the use of GCE that will benefit humankind worldwide will emerge very soon.

## Figures and Tables

**Table 1:** Orthogonal tRNAs derived from different species discussed in the review and their hosts of expression.

Host of Orthogonal tRNA	tRNA	Anticodons	Noncanonical Amino Acids	Host Organism	Refs
Archaeal					
*M. jannaschii*	tRNA^Tyr^	CUA	*O*-methyl-L-tyrosine	*E. coli*	[35]
	tRNA^Cys^	CUA	*O*-phosphoserine (Sep)	*E. coli*	[36]
	tRNA^Pyl^	UCCU	N-3-(tert-butyloxycarbonyl)-L-lysine (Boc-Lys)	*E. coli* and mammalian cells	[37]
	tRNA^Ser2^	UCCU	p-azido-L-phenylalanine	*E. coli*	[33]
*Methanosarcina. barkeri*	tRNA^Pyl^	CUA	*N^ε^*-acetyllysyl	*E. coli*	[38]
			*N^ε^*-D-prolyl-L-lysine and N-*N^ε^*-cyclopentyloxycarbonyl-L-lysine	*E. coli*	[39]
*Methanosarcina acetivorans*	tRNA^Pyl^	CGGA, CGGG	*p*-Nitrophenylalanine and 2-naphthylalanine	*E. coli* and rabbit reticulocyte cell-free systems	[40]
*Methanosarcina mazei*	tRNA^Pyl^	CUA	*O*-methyl-L-tyrosine	*E. coli*	[41]
			*N^ε^*-methyl-L-lysine	*E. coli*	[42]
				*Drosophila melanogaster*	[20]
			*N^ε^*-tert-butyloxycarbonyl-L-lysine	Mammalian cells	[20]
			*N^ε^* benzyloxycarbonyl-L-lysine	*C. elegans*	[43]
			*N^ε^*-methyl L-lysine		[44]
		UCA	*N^ε^*-tert-butyloxycarbonyl-L-lysine	*E. coli*	[45]
**Yeast**					
*S. cerevisiae*	tRNA^Tyr^	CCCG	2-naphthylalanine L-lysine	*E. coli*	[46]
	tRNA^Phe^	CUA	*p*-bromophenylalanine	*E. coli*	[47]
		CUA	*p*-nitrophenylalanine	*E. coli*	[48]
		ACCU, ACCG			[49]
		AGGG, AGAG			
		AUAG, ACCC			
		AGAG, CGGU			
		CGCU, CCCU			
		CUAU, GGGU			
**Bacterial**					
*E. coli*	tRNA^Tyr^	CUA	3-iodo-L-tyrosine	*E. coli*	[50]
				Mammalian cells	[51]
				*Drosophila melanogaster*	[52]
			3-iodo-L-tyrosine	Mammalian cells	[53]
				*S. cerevisiae*	
			4-azido-l-phenylalanine		
			*p*-acetyl-L-phenylalanine		
			*p*-benzoyl-L-phenylalanine		
			*p*-azido-L-phenylalanine		
			o-methyl-L-tyrosine		
			*p*-iodo-L-phenylalanine		
	tRNA^Leu^	CUA	*p*-azido-L-phenylalanine	*Schizosaccharomyces pombe*	[54]
			o-methyltyrosine	*S. cerevisiae*	
			α-amino-caprylic-acid		[55]
			o-nitrobenzyl cysteine		
*B. subtilis*	tRNA^Trp^	UCA	5-hydroxytryptophan	Mammalian cells	[56]
*Geobacillus stearothermophilus*	tRNA^Tyr^	CUA	*p*-methoxyphenylalanine (pMpa),	Mammalian cells	[57]
			*p*-acetylphenylalanine (pApa),		[58]
			*p*-benzoylphenylalanine (pBpa),		
			*p*-iodophenylalanine (pIpa),		
			*p*-azidophenylalanine (pAzpa)		
			*p*-propargyloxyphenylalanine		

**Table 2:** tRNA modifications in the anticodon stem in *Saccharomyces cerevisiae* and *Escherichia coli* and the respective modifying enzymes to be manipulated for the improvement of ncAA incorporation efficiency.

Anticodon Loop Position	*S. cerevisiae*	Enzymes	Refs	*E. coli*	Enzymes	Refs	Role during Incorporation
26	m^2^_2_G	Trm1	[106]	*-*	-	-	Improves translation fidelity efficiency
32	Cm	Trm7	[107]	Cm, Um	TrmJ	[108]	
	m^3^C, m^3^U	Trm140	[109]	s^2^C	IscS, TtcA	[110]	
	Ψ	Pus8	[111]	Ψ	RluA	[110]	
34	Cm, Gm, cmnm^5^Um	Trm7/Trm7	[107]	Cm, Um Cmnm^5^Um	TrmL TrmL	[108] [107]	
	ncm^5^U mcm^5^U	Elp complex Trm9	[112]	cmnm^5^Um	TrmL	[110]	
	m^5^C	Trm4	[113]	ac^4^C	TmcA	[110]	
	ψ	Pus1	[114]	mnm^5^se^2^U	TrmL	[110]	
	mcm^5^s^2^U	Uba4 Ncs2 Ncs6	[115] [116]	s^2^U	IscS TusA	[117] [110]	
	A → I	Tad2,3	[91]	A → I	TadA	[118]	
36	Ψ	Pus1	[119]	i^6^A	MiaA	[110]	
37	m^1^G	Trm5	[90]	m^1^G	TrmD	[110]	
				m^2^A	RlmN	[108]	
	m^1^I	Trm5	[120]	m^6^A	TrmN6	[110]	
				i^6^A	MiaA		
	yW	Tyw1-4	[121]	ms^2^i^6^A	MiaB		
				m^6^t^6^A	TrmO		
	I^6^A	Mod5	[122]				
38	Ψ	Pus3	[119]	Ψ	TruA	[110]	
40	m^5^C	Trm4	[113]	Ψ	TruA	[110]	

Legend: Cm, 2′-O-methylcytidine; m^1^G, 1-methylguanosine; Gm, 2′-O-methylguanosine; m^1^1, 1 methylinosine; ac^4^C, N^4^-acetylcytidine; ψ, pseudouridine; mcm^5^Um, 5-carbamoylmethyl-2′-O-methyluridine; i^6^A, N^6^-isopentenyladenosine; ms^2^i^6^A, 3-methylcytidine; 2-methyl-thio N^6^ isopentenyladenosine; yW, wybutosine, I, inosine; ac^4^C, *N*^4^-acetylcytidine; m^3^C, 3-methylcytidine; mcm^5^U, 5 carbamoylmethyluridine; mcm^5^Um, 5-carbamoylmethyl-2’-*O*-methyluridine; mcm^5^s^2^U, 5-methoxycarbonylmethyl-2-thiouridine; Um, 2’-*O*-methyluridine; m^5^C, 5-methylcytidine; *mnm^5^se^2^U*, 5-methylaminomethyl-2-selenouridine.

## Abbreviations

aaRS	aminoacyl-tRNA synthetase
ADC	antibody-drug conjugate
ASL	anticodon stem-loop
BCMA	B-cell maturation antigen
EF-Tu	elongation factor translation thermos unstable
EGFR	epidermal growth factor receptor
FPs	fluorescent proteins
GCE	genetic code expansion
GRO	genomically recoded organism
LXR	liver X receptor
MAGE	multiplex automated genome engineering
MjRS	methanocaldococcus jannaschii tyrosyl-tRNA synthetase
MoTTs	modification tunable transcripts
ncAA	noncanonical amino acid
NAAs	natural amino acids
ORF	open reading frame
OTSs	orthogonal translation systems
o-tRNA	orthogonal-tRNA
PACE	phage-assisted continuous evolution
PAcF	p-acetylphenylalanine
PTMs	post-translational modifications
RBP	retinol-binding protein
RF-1	release factor 1
RiPPs	ribosomally synthesized and post-translationally modified peptides
SCI	supplementation-based incorporation
SICLOPPS	split intein catalyzed ligation of proteins and peptides
URM1	ubiquitin-related modifier-1
